# Working memory and abstract thinking in patients with schizophrenic spectrum disorders

**DOI:** 10.1192/j.eurpsy.2021.1395

**Published:** 2021-08-13

**Authors:** M. Popov, I. Pluzhnikov

**Affiliations:** 1 Department For The Study Of Endogenous Mental Disorders, FSBSI «Mental health research centre», Moscow, Russian Federation; 2 Department Of Adult Neuropsychology And Abnormal Psychology, Moscow Institute of Psychoanalysis, Moscow, Russian Federation; 3 Department Of Youth Psychiatry, The Mental Health Research Center, Moscow, Russian Federation

**Keywords:** schizophrénia, neuropsychology, Working memory, Abstract thinking

## Abstract

**Introduction:**

According to a selective meta-analytical review, weakness of working memory is considered as one of the fundamental disorders in schizophrenia. Some researchers propose identifying this disorder as an endophenotypic marker of schizophrenia diathesis. Many researchers also emphasize violations of “abstract thinking”, that is, the ability of patients to operate with abstract concepts. Many scientists understand the violation of “abstract thinking” as the difficulty of patient in operating with the dominant signs of the concept. Based on these approaches, we assume a dark relationship between working memory and abstract thinking.

**Objectives:**

The aim of this study is to investigate the relationship between working memory and abstract thinking defect in patients with schizophrenic spectrum disorders.

**Methods:**

16 patients with schizophrenic spectrum disorders were studied. To study abstract thinking, the following neuropsychological and psychometric techniques were used: exclusion of objects, D-KEFS understanding of proverbs (latent concepts were recorded). The following techniques were used to study working memory: n-back; Wechsler Test, subtest Digit Repetition.

**Results:**

As a result of preliminary research, the following data was obtained. We found significant differences between the number of irrelevant features (which corresponds to impaired abstract thinking) and the severity of impaired working memory (p = 0.035). The more the memory was impaired, the more the subjects demonstrated the impairment of abstract thinking.

**Conclusions:**

Thus, our results justify our assumption. A relationship between working memory and abstract thinking is founded to be possible. Further studies of this issue requires a wider techniques battery as well as a larger sample.

**Conflict of interest:**

The reported study was funded by RFBR, project number 20-013-00772

